# Medical Observations on Certain Parts of the Delta of the Mississippi

**Published:** 1835

**Authors:** W. Kittredge

**Affiliations:** Locust-Grove, Assumption, La.


### XI.—Medical Observations on certain parts of the Delta of the Mississippi.

*Locust-Grove, Assumption, La., Sept.*, 1835.

Dr. Drake—Dear Sir,—In the last No. of your Medical Journal I noticed a communication from Dr. Cartwright, of Natchez, giving it as a fact, that "certain parts of the Delta of the Mississippi are proverbially exempt from autumnal diseases," or in other words, to the bilious fevers which other parts of said Delta are subject to. Now, from actual observation on the spot, I would beg leave to differ with the Dr. as to the fact of said exemption, and as to the cause of it, in case it did exist.

I will, however, admit, in the first place, that said section of country is comparatively healthy, with respect to some other portions, situated more interiorly; and much more healthy than might be supposed from its locality—being situated, as the Dr. justly remarks, on bayous without current, and surrounded by swamps and lakes, extending into bogs, that communicate with the Gulf of Mexico. But after admitting its comparative healthfulness, it is far from fact to say, it is not yearly subject, more or less, to bilious fevers of different grades, owing, of course, to difference in seasons, as hot and cool, wet and dry, etc. A wet summer throughout, for instance, is universally healthy; but a great quantity of rain at any time in the summer, followed by a few weeks of hot sultry weather, is as universally followed by severe bilious fevers.

In taking a view of the Dr's. position, we would be led to suppose that our autumnal diseases are dependent on the stagnant waters existing in lakes and bayous; and a vegetable growth on their surface, would make such localities perfectly healthy. This position I conceive to be utterly fallacious, and not tenable by fact or observation. As before observed, whilst the earth is kept constantly saturated with frequent showers of rain, and the swamps are kept full of water, we are always healthy: but when such state is succeeded by great and continued heat, drying away the water from the earth and swamps, fever is certain to succeed. This state of the atmosphere, *consequently* filled with miasmata, is not, I conceive, materially mitigated by the vegetable growth spoken of by the Dr. as covering the surrounding waters, as it is only one of nature's means of purifying *them*, and not the atmosphere any more than any other vegetable growth. The principal cause of the comparative healthiness of the region above alluded to, including the Bayou Lafourche, conceive to be, its contiguity to the Gulf of Mexico, and the constant prevalence of winds, mostly in a southerly direction, throughout the summer months.

I am sorry, therefore, to acknowledge, that even if the Dr. gets a vegetable growth to cover the lakes and bayous, in Concordia, I have my doubts, if the ills of humanity will be much mitigated thereby. It is not, however, at all probable, that they can be made to grow; on account of the overflown waters of the Mississippi coming in contact with the lakes and bayous, situated in that section, every spring.

I would make a single remark on another subject mentioned by the Doctor, viz: as to the best locality for persons predisposed to phthisis pulmonalis. I believe it is now generally admitted by the most enlightened of the profession, that localities situated immediately adjoining the sea, are not the best adapted for persons predisposed to this disease—more especially high and dry situations. The reason why I would reject such localities, is that the atmosphere is too inflammatory, if I may be allowed the expression, or too stimulating for the lungs of such patients. For this reason I would recommend the lowest and consequently the dampest situation that can be found, and as far south as possible, so as at the same time to be clear of the immediate influence of the sea breeze. Such locality can be found on the upper and middle portions of Lafourche—being in the latitude of New Orleans. From seven years observations I hardly known an original case of consumption.

Respectfully, your obd't servant,

W. Kittredge.

